# The evolving role of bariatric surgery in patients with type 1 diabetes and obesity

**DOI:** 10.15761/IOD.1000144

**Published:** 2016-02-15

**Authors:** Ali A. Rizvi

**Affiliations:** Department of Medicine, Director for Division of Endocrinology, University of South Carolina School of Medicine, USA

**Keywords:** obesity, type 1 diabetes, bariatric surgery, gastric bypass

## Abstract

Bariatric surgery has emerged as a viable treatment option in morbidly obese individuals with type 2 diabetes. Concomitant with societal lifestyle changes and the increased emphasis on achieving metabolic targets, there has been a rise in the number of patients with type 1 diabetes (T1DM) who are overweight and obese. Preliminary experience based on a limited number of observational reports points to substantial weight loss and amelioration of comorbid conditions such as blood pressure and dyslipidemia in patients with T1DM who undergo weight loss surgery. However, there is little evidence to suggest significant improvement in glycemic control and lowering of glycosylated hemoglobin, and bariatric surgical procedures do not necessarily lead to enhanced diabetes management. and improved quality of life. The potential possibility of micronutrient deficiency, weight regain, and psychobehavioral issues post-bariatric surgery also exists. An individualized evaluation of the risks and benefits should be considered, using a a multidisciplinary team approach with expertise in patient selection, surgical technique, and follow-up. A crucial component is the availability of a diabetes care specialist or endocrinologist experienced in intensive, tailored, modifiable insulin regimens who maintains close and careful monitoring during all phases of management. Reliable data from a prospective, longitudinal perspective is required to provide guidelines for clinicians and informed choices for obese patients with T1DM who are contemplating bariatric surgery.

## Introduction

Bariatric surgical procedures consist of either gastric banding or involve bypassing, resecting, or transposing portions of the stomach and sections of the small intestine [1]. The objectives of bariatric surgery (BS) are to reduce alimentary capacity, induce a malabsorptive situation, or both. When performed by a skilled surgeon and with input from a multidisciplinary team of professionals, metabolic surgeries can be effective weight loss treatments for severe and morbid obesity [2]. Ideally, the bariatric intervention should be part of a comprehensive weight management program with the availability of lifelong lifestyle support and medical monitoring [3]. Diabetes mellitus is representative of the major chronic health conditions that could be ameliorated or even remitted with the significant amount of weight loss and metabolic changes that occur after BS. Historically, BS has been used in patients suffering from type 2 diabetes (T2DM). Impressive weight loss and glycemic improvement, and even cessation of pharmacologic therapies, has been achieved [4]. In contrast, BS has not been utilized or studied to the same degree in type 1 diabetes (T1DM), and its advantages and drawbacks in this patient population remain to be fully elucidated.

## Bariatric surgery in patients with diabetes – a success story?

Over the past two decades, bariatric surgical procedures, particularly gastric bypass and duodenal switch, have been increasingly utilized in the management of obese individuals with type 2 diabetes (T2DM). Diabetes remission has been impressive in some reports; better success rates have been demonstrated with small intestinal bypass operations than with restriction of stomach capacity. The American Diabetes Association guidelines state that BS “may be considered for adults with body mass index (BMI) >35 kg/m^2^ and type 2 diabetes, especially if diabetes or associated comorbidities are difficult to control with lifestyle and pharmacological therapy” [4]. There is currently limited evidence for the benefits of BS in obese patients with BMI in the 30-35 kg/m^2^ range. In a Swedish study, remission of hyperglycemia was achieved and sustained two years after surgery in 72% of patients, compared with only 16% in a matched control group managed with lifestyle and pharmacological interventions, along with evidence of a reduction in mortality [5]. However, a Veterans Affairs population of obese subjects did not show mortality benefit after a mean follow-up of more than 6 years [6]. A study from the Cleveland Clinic evaluated the effectiveness of combined medical-surgical intervention compared to medical therapy alone in patients with uncontrolled T2DM; at 3 years the hemoglobin A1c (HbA1c) target of less than 6% was achieved by 38% in the those who underwent gastric bypass and 24% in the sleeve gastrectomy group, while only 5% in of those who received medical therapy achieved the same goal [7]. In general, T2DM patients with relatively shorter duration of disease and lower HbA1c, and those that are not using insulin preoperatively, tend to have better outcomes after BS [8].

The seemingly dramatic benefits of BS in patients with T2DM are tempered by the cost, risks, need for long-term lifestyle support and close medical monitoring, and the possibility of weight regain and nutrient deficiencies [9]. Other concerns include dumping syndrome (nausea, abdominal discomfort, and diarrhea) and severe hypoglycemia from hyperinsulinemia. However, surgical morbidity and mortality has decreased considerably in recent years; the 30-day mortality rates for laparoscopic procedures and open operations are 0.2% and 2.1% respectively [10]. Outcomes vary depending on the procedure and the experience of the surgeon and center. More recent studies also suggest that patients who undergo (BS) may be at increased risk for depression, suicide, and self-harm, including tobacco and substance use [11].

## Type 1 diabetes and obesity

Obesity is becoming more prevalent in patients with T1DM. The major reason behind this epidemiologic change is the increase in sedentary habits and dietary changes that is impacting all ages and societies. The latter is disproportionately affecting children and youth. It is estimated that the prevalence of overweight in young adults with T1DM ranges from 12.5% to 33.3% [12]. The increased emphasis on attaining glycemic goals and use of intensive insulin regimens has resulted in a higher rate of severe hypoglycemia and contributed to the propensity to weight gain. Thus, approximately 50% of patients with T1DM are currently obese or overweight, and between 8% and 40% meet the metabolic syndrome criteria [13,14].

**Figure 1:**
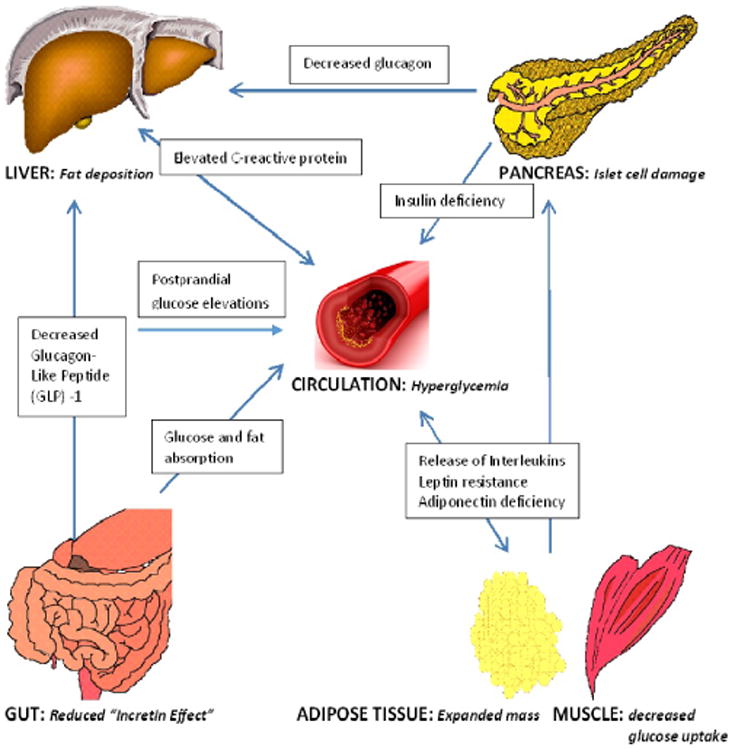
The interplay of hyperglycemia, hormonal pathways, and inflammatory markers in overweight and obese individuals with type 1 diabetes.

Figure 1 is a simplified depiction of the complex and multisystem hormonal and metabolic derangements that underlie the manifestations of hyperglycemia and inflammation in persons with T1DM. Insulin deficiency, elevated glucagon release, and progressive loss of amylin are the major pancreatic defects; derangements in incretins and gut hormones may contribute to post-prandial hyperglycemia, satiety issues, and problems with gastric emptying. The added burden of obesity and suboptimal glycemic control aggravates the pathophysiologic pathways in the liver and peripheral tissues that predispose to a proatherogenic environment. The components of the metabolic syndrome and insulin resistance have been linked to chronic T1DM complications, and cardiovascular disease is now the leading cause of death in these patients. Therefore, new therapeutic strategies are required in T1DM subjects, not only to intensively lower glycemia, but to control the associated metabolic syndrome traits [15,16].

## Data on Type 1 diabetic patients who have undergone weight loss surgery

A review of the literature reveals only a few reports of weight loss intervention procedures in patients with T1DM. The studies are mostly retrospective in nature and are limited by a small number of patients with heterogeneous characteristics. Table 1 lists the studies, with the number of subjects, results, and author conclusions. Although weight loss was achieved and insulin requirements were reduced considerably in many patients, the benefit with regard to glycemic control was highly variable and no uniform improvement was seen. Most of the authors concluded that the role of BS in T1DM warranted further research, particularly from the point of view of sustained improvement in glucose management.

Czupryniak and colleagues followed three female patients with T1DM who underwent gastric bypass surgery for a mean of 7 years after intervention [17]. Their impressive results on weight loss, glycemic control, and metabolic improvement contrast with the findings of 2 other studies [19,20] that did not show significant or sustained alleviation of hyperglycemia. A report from the Cleveland Clinic involving 10 morbidly obese patients with type 1 diabetes who had bariatric surgery (primarily gastric bypass) showed a reduction in the mean BMI from 41.6 to 30.5 and a decrease in mean A1c from 10% to 8.9% after a 3-year follow up [21]. Daily insulin requirements were reduced by almost half and beneficial changes in blood pressure and serum lipids were observed. The authors concluded that the favorable metabolic effects of BS may facilitate medical management of T1DM in the setting of morbid obesity, but that its true role awaited longer follow-up studies in a larger cohort. These inferences were felt to be overly optimistic by several groups of researchers who responded with their own experiences that failed to confirm glycemic benefits of BS in obese T1DM subjects [22-26]. Lanoo et al. speculated that sleeve gastrectomy may lead to a more predictable absorption of carbohydrates and might thus be a more attractive solution in the type1 diabetic population [26].

Recently published systematic reviews and meta-analyses generally point to T1DM patients attaining significant weight loss and resolution of comorbid health conditions with BS [27,28]. However, in spite of a decline in total insulin requirements, the reduction in glycosylated hemoglobin levels is modest at best, and overall glycemic control remains a challenge [29]. Additionally, although gastric bypass was the procedure most often utilized, the data was insufficient to recommend any particular type of intervention. The authors concluded that future studies needed to focus on more rigorous research methods and higher levels of evidence.

The conflicting findings with BS in T1DM with respect to successful weight loss and metabolic improvement on one hand and lack of predictable glucose lowering on the other also contrast with those in T2DM, thereby alluding to differing responsive mechanisms in the two conditions. The relative impact on gut physiology and the type of procedure utilized may both play a role. Malin and Kayshap [30] have highlighted the gut hormones as potentially important contributors to the durability of appetite suppression and countering of fat storage following BS. Table 2 provides an overview of BS in patients with type 2 and type 1 diabetes. Two recent reports focus on a comparison between bariatric intervention in type 1 and type 2 diabetes. Maraka and coinvestigators report the impact of weight-loss surgery on metabolic outcomes in ten patients with T1DM versus 118 patients with insulin-requiring T2DM [31]. Both groups lost similar amounts of weight 2 years post-bariatric surgery (39.5 ± 14.7 kg vs. 40.3 ± 24.4 kg). However, while T2DM subjects had significant improvements in HbA1c (7.8% ± 1.4% vs. 6.8% ± 1.4%, P value <0.0001) and reduction in antihypertensive and lipid-lowering medications, T1DM subjects did not exhibit a similar benefit (HbA1c 8.2% ± 1.6% vs. 7.8% ± 0.9%). The study reinforced the findings that improved glycemic control was not an expected outcome when considering BS in patients with T1DM, even though the desired weight loss was achieved successfully. In a 55-month data comparison of ten patients with T1DM matched with 20 with T2DM, the improvement in BMI was actually greater in the former (77.1% versus 68.3%, P value 0.14), while reduction in insulin requirements and remission in hypertension and dyslipidemia were similar; the effect on glycemic control in T1DM, however, was unimpressive [32]. The investigators suggested that that metabolic surgery for T1DM could be considered as a therapeutic option in selected obese patients to ameliorate components of the metabolic syndrome and lessen cardiovascular risk.

## Hurdles on the road ahead

The greatest challenge in translating the benefits of BS to individuals with T1DM is the lack of availability meaningful data (Table 3). Even though retrospective analyses and modeling studies suggest that BS may be cost-effective for diabetes, the results are largely dependent on assumptions about the long-term effectiveness and safety of the procedures [33,34]. Understanding the long-term benefits and risks of BS, especially in individuals withT1DM and in those who are not severely obese, will require well-designed clinical trials, with optimal medical therapy as the comparator. Unfortunately, such studies may not be feasible [35]. Both quantitative and qualitative evidence for BS in T1DM is limited. There is a need to prospectively study the impact of BS on body weight, insulin requirements, glycemic control, and metabolic outcomes in patients with suboptimally controlled T1DM. The data accumulated so far favors BS as a tool for weight reduction, but not necessarily glycemic improvement, in T1DM.

Another important but unanswered question is: how can we standardize intervention employing a consistent team approach in the selection, preparation, and monitoring of patients with T1DM who are candidates for BS? The latter involves both short-term and long-term follow-up, each phase presenting its unique issues. For example, patients with T1DM are predisposed to perioperative complications from autonomic neuropathy and hypoglycemia unawareness [36]. They are also at risk for the rapid development of severe hyperglycemia and diabetic ketoacidosis, thus demanding expertise and vigilance in the surgical and early postoperative phase [37]. Special attention to insulin requirements and fluid-electrolyte status may be warranted. Bariatric surgery teams that have traditionally been used to care for patients with T2DM may face challenges when encountering T1DM patients in similar settings. Input from diabetes educators and endocrinologists during all phases of management may be desirable. Table 4 summarizes the important points to keep in mind in the preparatory, pre- and perioperative, and postsurgical phases of bariatric surgery in patients with T1DM.

The choice of operation in patients with T1DM who are being considered for BS is unsettled. Data from T2DM subjects has affirmed the general superiority of bypass surgery over restrictive procedures. In one meta-analysis, gastric banding resulted in less one-year excess weight loss than sleeve gastrectomy and Roux-en-Y gastric bypass (33% vs. 70%) [38]. The improvement in hyperglycemia is thought to be multifactorial, involving caloric restriction, hormonal pathways, and the weight loss itself. However, similar mechanisms cannot be assumed to be operational in T1DM, keeping in consideration the different pathophysiologic underpinnings of the two conditions [30,31]. A near-lack of insulin-producing capacity and specific derangements in the incretin-immune system pathway in T1DM [32] may blunt the advantage from hormonal alterations conferred by BS. Additionally, T1DM patients may be prone to micronutrient and nutritional deficiencies because of autoimmunity and coexisting conditions such as celiac disease.

Since the major benefit of BS in T1DM is seen with weight reduction and metabolic changes, should weight-loss surgery be offered to well-controlled T1DM patients who are obese? An argument in favor of this approach would be betterment of risk factors and a possible reduction in cardiovascular morbidity and mortality – endpoints that have yet to be demonstrated. A lessening of the heightened pro-inflammatory burden seen in diabetic patients might be another reason in favor of an aggressive approach [40]. However, the possibility of unintended and deleterious consequences such as deterioration or unpredictability in glucose levels in previously well-controlled patients should be kept in mind.

## Conclusions

The traditional landscape of the overweight condition as a rare accompaniment of T1DM is changing rapidly. A global rise in the prevalence of obesity and the metabolic syndrome has not spared the type 1 diabetic population, and up to half of all individuals with T1DM may be overweight. BS has been successfully utilized by specialized centers in the management of obese patients with T2DM, and there seems to be no reason that those with T1DM should remain deprived of this benefit. However, current knowledge is insufficient to recommend these procedures routinely in T1DM, with its unique pathophysiology, and lack of reliable information on treatment response and long-term outcomes. Although weight loss and reduction in insulin requirements appear to be successfully achieved, sustained and meaningful improvements in glycemic control remain a challenge. In addition, the well-known concerns about nutrient deficiency, weight regain, and psychobehvioral risks in persons undergoing bariatric surgeries should be kept in mind. An individualized approach with realistic goals and expectations is necessary. Further studies devised in a prospective fashion are needed. Of paramount importance is the formulation of evidence-based guidelines for clinicians and patients that are optimized for best results but are modifiable as more knowledge is accrued.

## Figures and Tables

**Table 1:** A Summary of Studies on Bariatric Surgery in Obese Subjects with Type 1 Diabetes.

Ref.	Study	n	Gender F/M)	Type of surgery	Length of follow-up	Weight change	Change in insulin requirements	Glycemic control	Metabolic changes	Author conclusions
[17,18]	Czupryniak *et al.,* 2010	3	All female	RYGB	7 years	27-31% decrease	from 0.6-0.95 to 0.3-0.83 IU/kg	A1c ↓ 3-4%	Improved BP, lipids, micro-albuminuria	Recommend BS in T1DM
[19]	Raab *et al*., 2013	6	All female	RYGB=2 SG=1 BPDDS=3	unclear	BMI 37.3-46 to 25.8-29 one yr post-surgery	62-150 IU/day pre-, 15- 54 IU/day 1 yr post-surgery	A1c 6.7-9.8% pre-, 5.7-8.5% one year post-surgery	Not mentioned	Improvement in insulin sensitivity seen
[20]	Chuang *et al.*, 2013	2	1 male, 1 female	SG=1 RYGB=1	Not given	28% and 42%	↓ in male, insulin started in female	Unchanged at 8.8% and worsened from 6.3% to 10%	Improve-ments in lipids and sleep apnea	BS does not necessarily lead to improved glycemic control in T1DM
[21,22]	Brethauer *et al.,* 2014	10	9 female, 1 male	RYGB=7 GB=2 SG=1	Mean 36.8 months	Greater than 60% loss	Average reduction from 0.74 to 0.4 u/kg/day	Average A1c decreased from 10% to 8.9%	Favorable changes in lipids and BP	Sustained and significant benefits; may facilitate medical management of T1DM in obese; longer follow-up studies in a larger cohort needed
[23]	Tang *et al.,* 2014	6	All females	RYGB=1 GB=3 SG=2	variable	Average BMI reduction of 11.4	Reduction in all patients	Average A1c before surgery 8.1%, after surgery 8.2%	Not mentioned	Controversial; glycemic control may not improve in all obese T1DM patients
[24,25]	Middelbeek *et al.,* 2014	9	All females	RYGB=9	7.7 ± 5.8 weeks	Average BMI reduction of 11%	Daily insulin reduced by 38%	Average A1c reduction of 0.9%	Not mentioned	Effective for weight loss but not for glycemic control
[26]	Lannoo *et al.,* 2014	22	Not given	RYGB=16 SG=6	Mean 37 months	Average BMI 39.7 pre- versus 31.4 post-surgery	Daily insulin 92.5 pre-versus 48 units post-surgery	8.4 (8.0–8.9)% pre-versus 8.2 (7.8–8.6)% postsurgery	Not mentioned	Unable to confirm improvement in glycemic control
[27]	Ashrafan *et al.,* 2015	Review and meta-analysis	various	variable	BMI reduced by 11 kg/m^2^	Daily insulin decreased by 49 units	Average decrease in A1c: 0.93%	Improve-ments in BP and lipids	Results hetero-geneous, study quality low	
[28]	Mahawar *et al.,* 2016				decreased	reduction	Improved	Modestly improved	Glycemic control remains difficult	

A1c: hemoglobin A1c, BP: blood pressure, RYGB: Roux-en-Y gastric bypass, SG: sleeve gastrectomy, BPDDS: biliopancreatic diversion with duodenal-switch, GB: gastric banding

**Table 2:** A comparison of the effects of bariatric surgery in obese individuals with type 2 and type 1 diabetes.

Parameter	Type 2 Diabetes	Type 1 Diabetes
Weight loss	Yes	Yes
Decrease in diabetes medications/insulin	Yes	Reduction in insulin requirements, but insulin independence not possible
Improvement in glycemic control	Yes	Not conclusive
Possibility of disease remission (“cure”)	Yes	No
Improvement in blood pressure	Yes	Yes, but data is limited
Improvement in lipid profile	Yes	Yes, in small studies
Postsurgical changes in gut hormones contributing to metabolic improvement	Likely	Not seen or studied
Reduction in proinflammatory markers postprocedure	Yes	Not known
Development of postsurgical hyperinsulinemic hypoglycemia	Has been reported	Not reported
Superiority of gastric bypass over restrictive surgery	Yes	Unclear
Data on benefits and risks	Yes (still being gathered)	Not available

**Table 3:** Clinical Considerations for Weight Loss Surgery in Obese Individuals with Type 1 Diabetes.

Overall evidence supporting the effectiveness of bariatric intervention procedures in type 1 diabetes is limited; decision-making should be informed and individualized
Data is generally in favor of weight loss, reduced insulin requirements, and improvement in comorbidities (blood pressure and lipid profile)
Benefit it terms of glycemic control is not uniform; significant reduction in hemoglobin A1c has not been demonstrated in spite of successful weight loss; ease in daily glucose management may not ensue after surgery
A multidisciplinary patient-centered team approach is likely to deliver the best results; candidates should be carefully selected and should be aware of the benefits and risks of surgeryClose and careful follow-up in the hospital and during long-term care is essential
Because of the unique specialty expertise required for the optimal management of patients with type 1 diabetes, it is advisable to involve an experienced diabetes specialist or endocrinologist before, during, and after bariatric surgery
Post-bariatric surgery, vigilance should be maintained regarding optimizing nutrition, minimizing weight regain, and screening for psychological issues; patient enrollment in a life-long support group should be considered
Prospective studies are needed to evaluate the durable impact of surgical weight loss interventions on quality of life and treatment satisfaction in individuals with type 1 diabetes

**Table 4:** Important Aspects in the Medical Care of Patients with Type 1 Diabetes undergoing Bariatric Surgery.

**In the selection and preparatory phase, the patient**	Is following a prescribed lifestyle and instructions
	Has attended formal bariatric surgery and diabetes education classes
	Is motivated and adherent to diabetes self-management
	Has been optimized on a multi-dose insulin injection (MDII) regimen or continuous subcutaneous insulin infusion (CSII, or insulin pump)
	Understands the benefits and risks of bariatric surgery
**Before the surgical procedure**	Administer half to two-thirds of the usual dose of basal insulin the morning of surgery, omit bolus or rapid-acting insulin while fasting
	Start D5/W drip at 100 ml/hour
	Start continuous intravenous insulin (insulin drip) 2-3 hours prior to surgery
	Check hourly fingerstick readings and maintain blood glucose level between 140 to 180 mg/dl
**During the surgical procedure**	Maintain D5/W and insulin drips
	Measure glucose levels every hour and adjust insulin drip rate to maintain readings between 140 to 180 mg/dl
**In the postoperative phase**	Continue D5/W and insulin drips and monitor glucose levels hourly until the effects of anesthesia and any nausea or vomiting has subsided
	Maintain attentiveness to fluid and electrolyte status in the intensive care or step-down unit
	Watch for early postsurgical complications (infection, wound dehiscence, etc.)
	Resume basal and preprandial bolus insulin as early as feasible when gradual oral intake is resumed, and modify as new dietary habits are established
**During follow-up**	Plan discharge to include nutrition and behavioral counseling and adjustment of insulin regimen
	Make early (within 1-2 weeks) post-surgery appointment in the office/clinic to review nutrition, glycemic status, need for adjustment of insulin, and management of comorbid conditions
	Monitor long-term for late complications of surgery, metabolic abnormalities, micro- and macronutrient intake, prevention of weight regain, and behavioral issues
